# Moderated online social therapy for carers of young people recovering from first-episode psychosis: study protocol for a randomised controlled trial

**DOI:** 10.1186/s13063-016-1775-5

**Published:** 2017-01-17

**Authors:** John Gleeson, Reeva Lederman, Helen Herrman, Peter Koval, Dina Eleftheriadis, Sarah Bendall, Sue M. Cotton, Mario Alvarez-Jimenez

**Affiliations:** 1 School of Psychology, Faculty of Health Sciences, Australian Catholic University, Fitzroy, Australia; 2 The Department of Computing and Information Systems, The University of Melbourne, Parkville, Australia; 3 Orygen, the National Centre of Excellence in Youth Mental Health, Melbourne, Australia; 4 Centre for Youth Mental Health, The University of Melbourne, Melbourne, Australia; 5 Research Group of Quantitative Psychology and Individual Differences, Faculty of Psychology and Educational Sciences, KU Leuven, Leuven, Belgium

**Keywords:** First-episode psychosis, Carers, Family intervention, eHealth, Randomised controlled trial

## Abstract

**Background:**

First-episode psychosis most often has its onset during late adolescence. In caring for the young person, families endure high levels of stress and depression. Meanwhile, the social networks of families often erode. Our group has previously shown that family cognitive behaviour therapy (CBT) leads to significantly improved perceived stress compared with specialist first-episode treatment as usual; however, there are well-known barriers to the dissemination of effective family interventions. To address this, we have developed a novel online intervention entitled ‘Altitudes’ that fully integrates purpose-built online social networking, expert and peer moderation, and evidence-based psychoeducation within a single application. The primary aim of this trial is to evaluate the effectiveness of Altitudes in reducing stress in carers over a 6-month period.

**Methods/design:**

We describe here a single-blinded cluster randomised controlled trial (cRCT) with permutated blocks. The clusters comprise individual families. The two treatment conditions include Altitudes plus Specialist Treatment as Usual (STAU) and STAU alone. Altitudes involves participation in our novel online programme whereas STAU comprises specialist family work at the Early Psychosis Prevention and Intervention Centre (EPPIC), Melbourne, Australia. We aim to recruit 160 family members of young, 15–27 year-old, patients registered for treatment for first-episode psychosis (FEP) at EPPIC. The design includes two assessment time points, namely, baseline and 6-month follow-up. The study is due for completion within 2 years including an 18-month recruitment period and a 6-month treatment phase. The primary outcome is carers’ perceived stress at 6 months. Secondary outcome measures include a biomarker of stress, depressive symptoms, worry, substance use, loneliness, social support, satisfaction with life, and a range of measures that tap into coping resources. We seek to gain a dynamic picture of carer stress through our Smartphone Ecological Momentary Assessment (SEMA) tool.

**Discussion:**

This is the first randomised controlled trial designed to evaluate an online intervention for carers of young people recovering from FEP. It has the potential to produce evidence in support of a highly novel, accessible, and cost-effective intervention to reduce stress in carers who are providing support to young people at a critical phase in their recovery from psychosis.

**Trial registration:**

Australian New Zealand Clinical Trial Registry, identifier: ACTRN12616000968471. Retrospectively registered on 22 July 2016.

**Electronic supplementary material:**

The online version of this article (doi:10.1186/s13063-016-1775-5) contains supplementary material, which is available to authorized users.

## Background

Psychosis is a severe mental disorder, which impacts not only the individual but also their family. Family members caring for a relative with psychosis often suffer significant levels of distress [1]. Fortunately, the acute symptoms of psychosis, which most often have their onset in late adolescence, typically respond to treatment [2]. However, full recovery of functioning is a longer-term process taking several years in many cases [3]. Young people recovering from first-episode psychosis (FEP) typically face elevated risks of depression [4], social anxiety [5], substance abuse [6], and long-term challenges in returning to meaningful activities [7]. Forty percent of first-episode patients suffer psychotic relapse over the first 2 years after treatment is commenced [8].

### The experience of caregiving

Family members caring for the young person with FEP also suffer from a range of significant physical and mental health problems. Investigations into the experience of caring for a relative have revealed that throughout the early stages of psychosis, family members have a significant involvement in a young person’s life; 80–90% of young FEP service consumers reside in the family home [9]. The commencement of acute-phase treatment for psychosis is often overwhelming for families and has been compared to bereavement [10]. During this period family members face the risk of exposure to traumas, such as violence perpetrated by the young person [11, 12], and families worry frequently about behavioural changes [13]. Twenty-six per cent of FEP family carers experience severe stress and a similar proportion suffer from moderate stress [14]. Depression is also common among family carers, with one third suffering clear depressive symptoms and another third mild depression [15]. Meanwhile, the burden of the caring role has been shown to significantly deplete family social networks, an important buffer for stress [16]. Although less is known about the physical health correlates of caring for a relative with psychosis, there is evidence that the style of caring and severity of psychotic symptoms increases the risk of physical health problems for relatives [17, 18]. Findings from investigations of Alzheimer’s disease provide a basis for further hypotheses – studies have suggested that stress is associated with weakening of carers’ immune functioning and accelerated ageing [19].

### Carers’ appraisals and the course of psychosis

We previously demonstrated that positive reappraisals by carers promote carer coping and reduced distress [20]. Carers’ appraisals of their relative’s disorder are also known to influence their communication with their relative. For example, if family members blame themselves for the psychosis they are significantly more likely to become emotionally over-involved with their relative [15]; we have shown that emotional over-involvement predicts burden of care and stress levels in families [21]. These are doubly important processes because these family communication patterns influence the course of psychosis – in particular, criticism and emotional over-involvement significantly increase the risks of psychotic relapse [22]. Therefore, for the mental health of all family members, it is critical to identify effective ways of reducing carers’ stress by encouraging positive reappraisal and by increasing the capacity of the family to respond adaptively to the young person affected by psychosis.

### The evidence for the effectiveness of family interventions for psychosis

Meta-analyses of randomised controlled trials (RCTs) of carer interventions in chronic psychosis have shown robust beneficial treatment effects leading to reduced rates of relapse in patients diagnosed with psychosis [23]. There is emerging evidence that carers also directly benefit from targeted interventions [24] and such interventions have been proven to be cost-effective [25]. These treatments are typically based upon cognitive behaviour therapy (CBT) with a focus on psychoeducation and training in structured problem solving and communication skills. Family interventions have also been structured to cater for multiple families simultaneously (i.e. multifamily therapy) with the added component of encouraging support between families [26].

Correctly targeted family intervention in the early stages after FEP may alter the long-term trajectory of family stress [27] and may improve the long-term outcomes for the young person [28]. The evidence base for the effectiveness of family interventions in FEP, however, is less well developed than for chronic psychosis [9].

Our group has published one of the few RCTs designed to evaluate an intervention specifically developed for FEP families whose relatives were receiving treatment at the Early Psychosis Prevention and Intervention Centre (EPPIC), a formative specialist FEP service in Melbourne, Australia [27]. In one treatment arm of the 30-month follow-up study, families and their young relative diagnosed with FEP received parallel CBT. This package of interventions, provided over a 7-month period, was compared with ‘gold-standard’ specialist treatment as usual (STAU) within a FEP service, and not generic ‘usual care’. Perceived stress related to caregiving was significantly improved in the family CBT condition compared to STAU at 30 months’ follow-up. In a separate study conducted at EPPIC, McCann and colleagues showed that FEP families randomised to self-guided bibliotherapy, a treatment based on problem-solving therapy, showed more favourable outcomes on positive caregiving experiences compared with those receiving STAU at 16 weeks’ follow-up [29].

Despite these promising results, a major gulf remains between the efficacy and the availability of family treatments in FEP. This gap is most evident for regular interventions or supportive structures for the families of all FEP patients, as well as being a concern for more complex cases where expert support is needed [30–32]. Evidence-based interventions are perceived as costly with the result that most families do not receive them. Where family interventions are available they are typically focussed upon the prevention of relapse and not on the wellbeing of the family members.

In this context the Internet may prove an important cost-effective and accessible resource for both online social support and for the provision of expert evidence-based interventions. The social isolation and vicarious stigma experienced by families, and the high value placed on opportunities to share concerns and effectively solve problems with other families [33], suggest that safe and structured online social networking may provide benefits. Online social networking offers the promise of high cost-effectiveness, wide dissemination, optimal engagement, and high accessibility that extends well beyond the limitations of the clinic setting.

Despite these possibilities we are aware of only two published studies in the psychosis field that have utilised information communication technology for carers, which involved variations of social networking [34, 35]. Neither study was designed specifically for FEP carers, and only one was an RCT [35]. In brief, these studies have demonstrated the acceptability of online interventions for carers of individuals with schizophrenia but have not provided specific evidence for the efficacy of online intervention for FEP carers.

We have successfully developed and piloted a new carer intervention entitled ‘Altitudes’ for carers with a young relative recovering from psychosis, after previously piloting the system with carers of young people with depression and anxiety. Altitudes integrates purpose-built online social networking, expert and peer moderation, and evidence-based psychoeducation within a single application. Altitudes was developed using participatory design principles in consultation with carers and with family peer-support staff members at Orygen Youth Health (OYH).

Altitudes is one of several applications derived from our Moderated Online Social Therapy (MOST) software framework. MOST was developed with a multidisciplinary team of clinical psychologists, psychiatrists, web designers, computer programmers and professional writers. Other examples of MOST applications include ‘Horyzons’ which was built specifically for service consumers with early psychosis [36].

We believe that Altitudes provides a unique opportunity to extend the availability of evidence-based family intervention for FEP families. Here, we describe the protocol for the Altitudes trial.

### Aims and hypotheses

The overall aim of this trial is to determine the effectiveness and cost-effectiveness of Altitudes for carers’ of young people with FEP. The primary hypothesis is that Altitudes plus specialist first-episode family treatment as usual (STAU), compared with STAU alone, will be superior in relation to carers’ perceived stress at 6 months’ follow-up. STAU was selected to test whether Altitudes affords improved outcomes in comparison with current best practice in FEP. Second, we hypothesise that Altitudes plus STAU will be superior to STAU at 6 months’ follow-up in relation to an objective measure of stress, carers’ depression, carers’ self-efficacy, and carers’ perceived social support.

A supplementary aim of the study is to understand the mechanisms underpinning stress in carers. A major innovation of this trial is the successful creation of a cutting edge Smartphone Ecological Momentary Assessment (SEMA) application. Using this technology, we aim to investigate how variance in carers’ stress and depression over time is accounted for by: (1) the nature of specific interactions with their young relative, (2) the content and level of conviction associated with specific appraisals of their relative’s behaviour, (3) the appraisal of emotional and social support from others, (4) specific coping responses to the young person’s behaviour, and (5) their perceived self-efficacy in caring for their young relative. Importantly, SEMA allows us to measure these potential mechanisms of therapeutic change in real time as they naturally occur in carers’ daily lives.

## Methods/design

### Study design

A cluster randomised controlled trial (cRCT), with families comprising clusters, is designed to test the effectiveness of Altitudes plus STAU (based upon our MOST software) compared with currently available STAU in improving stress at 6 months in carers of young people recovering from FEP.

The design includes two assessment time points, namely, baseline and 6 months’ follow-up with assessors kept blind at each time point. An additional interim interview is completed via telephone at approximately 3 months after the baseline assessment to assess perceived stress.

This cRCT includes an 18-month recruitment period and a 6-month treatment phase. The study will be completed within 2 years. The protocol’s development was guided by the Standard Protocol Items: Recommendations for Interventional Trials (SPIRIT) (Additional file 1) [37] and Good Clinical Practice guidelines [38].

### Setting

Recruitment of the trial participants commenced in October 2015 at EPPIC, a subprogramme of Orygen Youth Health (OYH). EPPIC is a specialist FEP programme that provides services to 250 new FEP patients per year [39]. EPPIC provides 18 months to 2 years of specialised care after which patients are discharged and transferred to standard treatment if required [40].

### Participants

Eligible participants include carers (namely parents, grandparents, siblings, and partners) of young people (aged 15–27 years inclusive) who: (1) are currently receiving treatment for FEP at EPPIC or (2) have recently been discharged by their treating team after an episode of care at EPPIC. There is no specified limit on the number of eligible participants from each family. Clients eligible to be admitted to EPPIC services: (1) have a diagnosis of a first episode of a . *Diagnostic and Statistical Manual of Mental Disorders* (*4th ed.*), *text revised* (DSM-IV-TR) [41] psychotic disorder or mood disorder with psychotic features, (2) are aged 15–25 years inclusive, and (3) have had at least 6 months’ treatment with an antipsychotic medication prior to registration with EPPIC.

### Enrolment and randomisation

**Fig. 1:**
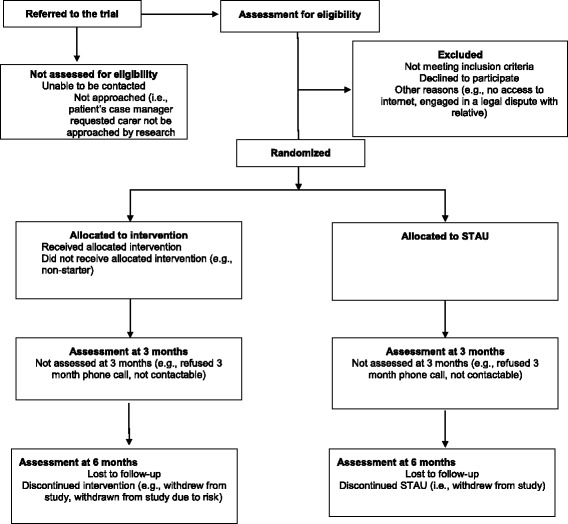
Study flow diagram for Altitudes

The recruitment and allocation is depicted in the flow diagram in Fig. 1. The study research assistants (RAs) regularly attend EPPIC clinical team meetings to promote the study. After the RA obtains written informed consent from each family member, families are randomised to Altitudes plus STAU or to STAU alone at a ratio of 1:1. We expect that participant numbers within clusters (i.e. families) are likely to vary from between one and four with a mean cluster size estimate of 1.3. An independent statistician created the randomisation sequence, which includes permutated blocks. The block sizes and randomisation sequence are concealed from the study RAs and investigators. Randomisation occurs after each baseline assessment. When a family member from a newly recruited family provides informed consent and completes the baseline assessment, the study coordinator randomises the family via a secure online Clinical Trials Management System (CTMS). The CTMS sends an automated email to the study coordinator and investigators notifying them of the outcome of randomisation. The study coordinator informs the family of the allocation.

The study RAs undertaking the follow-up assessments are kept blind to treatment allocation via the following mechanisms: (1) regular reminders are sent to all clinical staff at EPPIC regarding the importance of the blind, (2) at the commencement of each research interview the RA reminds participants of the importance of the blind, (3) study RAs are excluded from all clinically related discussions regarding participants, and (4) the RAs are forbidden from accessing participants’ medical records. The RAs record their best guess of participants’ treatment allocation at 6 months’ follow-up in order to enable an assessment of the success of treatment concealment.

### Interventions

We designed and built Altitudes utilising our MOST software framework which includes three highly integrated functions within one web-based application. These include: (1) online psychoeducation and interactive therapy (divided into specific thematic pathways which are further separated into individual ‘steps’), (2) expert-moderated social networking (via a ‘café’), and (3) peer moderation. The content and functionality of the application was informed by multiple group consultations sessions with carers. Each user can log on at their convenience at any time.

#### The features of the online interactive psychoeducation

The study coordinator inducts new users into Altitudes by orientating them to the system, providing passwords, and highlighting ways to optimise its use and ways to access system help. Users complete, at their own convenience, a series of ‘pathways’ organised into distinct themes including self-care, understanding psychosis, early warning signs and prevention of relapse, understanding their personal strengths as a carer, managing behavioural problems in their relative, and communicating with their relative. In order to maximise the usability of the material, these pathways are comprised of brief thematically related psychoeducation and interactive therapy ‘steps’. The content of these steps has been specifically designed to improve carer stress, e.g. by encouraging self-care, by facilitating carer self-efficacy, and by targeting problematic appraisals known to increase carers’ stress. In addition, the content of the steps have been influenced by social cognition concepts of agency and self-efficacy in family life [42]. The steps and pathways entail regular prompts to users to share their reactions to material with other users through a series of ‘talking points’. To ensure that psychoeducation is translated into meaningful behavioural change, we built specific actions entitled ‘do its’, which are related to therapy content (e.g. to practice mindfulness exercises) and to users’ specific strengths. Users’ comments on steps and completed actions populate the content of the social networking newsfeed. In addition, users are able to indicate their preference for material through ‘like’ buttons, rate the relevancy of content, share content with others users, keep track of which users have completed specific pathways, and identify other users who share their personal strengths. Users can also utilise a ‘team up’ function to indicate their support or join other users in striving for specific behaviour change.

#### The social networking features

Users of Altitudes are encouraged to communicate with each other and with moderators through the online ‘café’ where all other users are visible in the ‘network’ page. Expert moderators (clinical psychologists) are identifiable as a separate class of users within the network. Users can visit the ‘wall’ of fellow users where posts (and comments upon posts) are displayed along with profile information and images uploaded by each user. Moderators can promote specific content within each user’s home page, including suggested actions, which moderators select based upon individual users’ strengths and motivations to use the system. The café menu also includes a group problem-solving function, entitled ‘talk it out’, derived from moderated problem solving as outlined in multifamily therapy research [43]. Users can suggest everyday problems in caring for their relative, and moderators invite other users to join in the ‘problem-solving group’. The system stores previous problems and solutions, providing an easily accessible ‘solution wiki’ to subsequent users. The social networking combined with problem solving and psychoeducation has been designed to provide social support, increase carers’ understanding of their relative’s disorder, and increase flexibility of interpersonal problem solving and communication.

#### Altitudes workshops

Moderators invite users to Altitudes ‘meet ups’, offline face-to-face gatherings held approximately every 6 months during the course of the trial. The Altitudes meet ups are designed to provide users with the opportunity to ask the expert moderators questions and give feedback on the system and encourage increased online interaction between users and moderators of Altitudes.

#### The role of online moderators

Expert moderators, who are clinical psychologists with specialist family work experience, log on to Altitudes at daily intervals with the goal of monitoring safety and encouraging self-care, self-efficacy and positive coping within families by making comments on the newsfeed and sending direct messages to users [42]. Peer moderators, with lived experience of caring for a relative with psychosis, model the use of the system and facilitate online interactions. Moderators present formulations of users at weekly supervision sessions including analyses of system activity and planned interventions to optimise users’ engagement, increase positive affect, and address any specific clinical need by suggesting content matched to users’ interests and needs. Through analytics available on the back-end moderator interface, moderators identify users who are at risk of reduced engagement and form a follow-up plan, which might include prompts to log on or suggestions about the content of Altitudes that matches with the users’ identified needs and interests. Our approach to online moderation was inspired by the Supportive Accountability framework of eHealth interventions, which highlights that human support is critical to ensuring a necessary level of engagement in eHealth systems [44]. Supportive Accountability incorporates self-determination theory, which accounts for individual differences in motivation in terms of the fundamental human needs of relatedness, competency, and autonomy. Moderators develop individualised formulations regarding users’ level of motivation to use Altitudes and how their specific needs can best be addressed to optimise system engagement [45].

#### Specialist family treatment as usual

All trial participants have access to the usual array of services for carers at EPPIC. This includes access to: (1) a series of three group-based, evening psychoeducation sessions entitled ‘Family and Friends’, (2) psychoeducation and support provided by EPPIC outpatient case managers, and (3) specialist family therapist sessions in specific cases, e.g. a brief course of face-to-face family sessions where the young person suffers from a high level of behavioural disturbance.

### Outcome measures

#### Primary outcome

The primary outcome of the trial is carer perceived stress measured by The Perceived Stress Scale (PSS) [46] 3 and 6 months after treatment allocation. The PSS measures perceived stress over the preceding month. The PSS is a valid and reliable 10-item measure rated on a Likert scale ranging from 0 (never) to 4 (very often).

As shown in Table 1, primary and secondary outcomes are measured prior to randomisation and at 6 months’ follow-up. A telephone interview is conducted at 3 months to collect interim ratings on perceived stress by completing The PSS with the participant via telephone. In addition, carers’ momentary appraisals and coping efforts and interactions between carers and their relatives will be tracked over 1 week as they naturally occur in daily life using SEMA, at baseline and 6-month follow-up.

**Table 1:** Schedule of outcome measures

	Time point (months)		
Measure	Baseline	3	6
Primary outcomes			
The Perceived Stress Scale	X	X	X
Secondary outcomes			
Hair cortisol	X		X
Centre for Epidemiologic Studies Depression Scale-Revised (CESD-R)	X		X
Penn State Worry Questionnaire (PSWQ)	X		X
Alcohol, Smoking and Substance Involvement Screening Test (ASSIST)	X		X
UCLA Loneliness Scale	X		X
‘Me as a Parent’ Questionnaire	X		X
Ways of Coping Scale (WOC)	X		X
Medical Outcomes Study: Social Support Survey (MOS-SSS)	X		X
Strengths Use Scale (SUS)	X		X
Self-Compassion Scale Short Form (SCS-SF)	X		X
Mindful Attention Awareness Scale (MAAS)	X		X
Satisfaction With Life Scale (SWLS)	X		X
Smartphone Ecological Momentary Assessment (SEMA)^a^	X		X
Family level measures			
The Family Questionnaire (FQ)	X		X
Experience of Care-giving Inventory (ECI)	X		X
The Parent-Adolescent Communication (PAC) Scale	X		X
Subsidiary measures			
AQoL 8D questionnaire	X		X
Resource Use Questionnaire	X		X
Altitudes-specific measures			
Altitudes Perceived Competence Scale	X		X
Altitudes Self-Regulation Questionnaire (A-SRQ)	X		X
Altitudes Health Care Climate Questionnaire (A-HCCQ)	X		X

^a^Smartphone Ecological Momentary Assessment surveys

#### Secondary outcomes

Hair cortisol is a biomarker of chronic stress [47] via long-term alterations in basal hypothalamic-pituitary-adrenal (HPA) axis activity [48]. Mean baseline HPA system activity during the last month is measured by a validated procedure for measuring hair cortisol [48]. A single hair sample (3 cm long, approximately 0.5 cm in diameter) will be collected by the RA from a posterior vertex region on the head. It will be cut from a 0.5-cm patch using scissors as close to the scalp as possible. The hair samples will be wrapped in aluminium foil for protection and stored in plastic tubes. The PSS has been commonly used in conjunction with hair cortisol measurements [49].

Secondary outcomes also include severity of depressive symptoms [50], worry [51], substance use [52], and loneliness [53]. A range of measures tapping into resources to cope with stress include parental self-efficacy [54], coping [55], social support [56], strengths’ use [57], self-compassion [58], and mindfulness [59]. Satisfaction with life will be assessed by the Satisfaction With Life Scale (SWLS) [60].

In order to gain a more dynamic picture of carer stress participants utilise our novel SEMA tool. The SEMA tool is readily downloadable at no charge to participants possessing a smartphone (running Android or iOS operating systems). The tool delivers surveys (administered following the baseline and 6-month assessment time point) at eight time points per day during the waking hours of each participant for a period of 1 week. Participants are prompted to complete SEMA surveys every 90 min (±30 min) over a 12-h period (e.g. 9 a.m. to 9 p.m.) each day for seven consecutive days. SEMA tracks participants’ responses in (near) real time, and ensures minimal loss of data by uploading responses to a secure server or caching responses on the users’ smartphone when an Internet connection is temporarily unavailable. The survey questions include items regarding any interaction with the young person since the last survey (e.g. ‘How much time have you spent interacting with your relative since the last survey?’), perceived behavioural problems in the young person (e.g. ‘My relative has irritated me since the last survey’), attributions for perceived behavioural problems (e.g. ‘I have felt my relative let me down by their behaviour since the last survey’), self-reported coping strategies (informed by our factor analysis of carer coping) (e.g. ‘I have reminded myself that things will work out in the end’) [20], perceived parental self-efficacy at the point of the survey (e.g. ‘During the last interaction, did you handle your relative’s behaviour well?’), perceived stress and depression (e.g. ‘At the moment I feel sad’), and perceived social support (e.g. ‘I have received support or encouragement from others since the last survey’).

Cost-effectiveness as measured by the AQoL 8D questionnaire [61] which allows for the calculation of Quality*-*adjusted Life Years (QALYs) at 6 months’ follow-up. In addition, a Resource Use Questionnaire (RUQ) is used to determine the broader resource use of participants. Additionally, for consenting participants, information regarding utilising of primary health care services will be accessed from Commonwealth Government authorities.

#### Patient and family characteristics

The mental health status of the young person, including their primary and comorbid problems are collected at baseline from carers and is verified against medical records if the young person provides consent.

Carer demographic variables recorded at baseline include age, living situation, years of education completed, employment and marital status, country of birth, and source of income.

Given that families are the unit of randomisation in the current study, relevant family level variables are measured to investigate whether they are independent from individual-level treatment effects. This is especially relevant given the nature of the social networking component of the intervention which may result in interfamily communication effects. Therefore, we include a measure of expressed emotion [62], the impact of the illness on the family [63], and the degree of openness and extent of problems in family communication [64].

#### Altitude-specific measures

Usage of the Altitudes online system is continuously monitored across the study intervention period (i.e. frequency, duration, and patterns of use). In addition, users of Altitudes complete self-report measures of their perceived competence using Altitudes [65], motivations for using Altitudes [66] and their perception of moderation by Altitudes [67].

### Safety

The safety protocol comprises three levels of security including: (1) *system and privacy protection*, (2) *online safety*, and (3) *clinical safety* [68].

The application is hosted on a University of Melbourne web server. The University has standard measures in place to prevent unauthorised access to the server. In addition, the web application includes measures to secure the application and database against unauthorised access. These measures conform to industry best practice as defined by the Open Web Application Security Project [69].

We manage privacy and online safety in accordance with the Australian Communications and Media Authority (ACMA). Temporary or permanent withdrawal from Altitudes is triggered by more than one incident of inappropriate use of the system.

The study coordinator conducts an individual face-to-face induction with Altitudes participants, including details of the terms of use. Altitudes includes a ‘report function’, which enables users at any time to indicate to moderators concerns about any inappropriate material posted by a user. The moderator assesses the basis of the report and responds accordingly, which can include the removal of the material and, in some cases, the deactivation or restriction of the user’s account. In addition, users can ‘switch off’ their profile and hide all of their existing comments on the system should they become concerned about their privacy. If participants do not comply with the guidelines for safe use of Altitudes they can be excluded from the system.

In addition to appropriate use of the system, moderators screen the system daily for evidence of clinical risk, including reports of evidence of deterioration in the mental state of the young person in the care of participants. Where indicated, the moderator conducts a risk assessment based upon available information and advises the participant on appropriate action, including accessing services. The system incorporates visible emergency guidelines and contact information.

Altitudes includes an automated keyword function that is activated each time a participant posts a contribution containing potentially offensive words. The function blocks posts with notifications sent to the user and the Altitudes moderator who can decide to ‘unblock’ the post.

### Data integrity

A custom-built Clinical Trials Management System (CTMS) is used to manage the electronic data of this study. The CTMS includes an electronic Case Report Form (eCRF) and randomisation functionality. The study RAs record participant-level data on a paper-based Case Report Form (CRF). These data are subsequently entered into the eCRF section of the CTMS. The randomisation functionality of the CTMS is used to carry out the randomisation aspect of the study and is operated by the study coordinator. The CTMS is accessed using a secure website and is stored on a secure server. It is designed to maintain the privacy and confidentiality of participant information and to ensure the integrity of the data. Access to CTMS is restricted to study personnel and the level of access is dependent on the person’s role. In particular, the RAs and the investigators do not have access to the randomisation section of the CTMS to ensure that they remain blind. Data are accumulated on three separate secure computer servers, including data collected from the SEMA tool, CTMS and data accumulated from participant activity within the Altitudes online system. Data are also provided from the laboratory analysis of hair samples at the Stratech Scientific APAC laboratory. These various data are aggregated into a single electronic secure databank.

### Statistical analyses and sample size

Between-group differences in the primary outcome will be examined using mixed-model repeated measures (MMRM) which are the preferred methods for the analysis of clinical trial data in psychiatry [70]. Individual time-point measures can be considered to be nested within carers, who may also be considered to be nested within families. MMRM will be used for the analysis of primary and secondary outcome measures. MMRM enables analysis of hierarchically structured data (e.g. allows for violations of assumptions such as homogeneity of regression slopes across time points and effects at the family level) while allowing maximum flexibility in the case of missing data [70].

We have assumed that moderate to large effect sizes will be obtained for the primary outcome of stress. We estimate, based on previous trials including carers at EPPIC, that the cluster size will be small (approximately 1.3) because, in most cases, there will only be one family member involved. An approximate estimate of the intracluster correlation for stress measures is .20. In most cases this would be the two parents/partners which would minimise the influence of genetic effects. In addition, patients’ symptoms account poorly for variance in carer measures of stress, so the estimation of intracluster correlation may even be inflated. Therefore, we estimate that the design effect will be approximately .26.

For continuous measures, if we set *α* at 0.05, a sample size of 64 is required for each of the two groups (total *n* = 128) to achieve power (1 − *β*) of 0.80. A study by Rotondi and colleagues (2010), which included randomisation to a multifamily web-based intervention reported a dropout rate of 3% at the 12-month follow-up, highlighting the acceptability of the design [35]. Taking a more conservative estimate and assuming an attrition rate in the proposed study of approximately 20% at follow-up, we have aimed to recruit 160 family members at baseline to retain 128.

## Discussion

The current trial aims to build upon our previous work in developing specialist family based interventions for families affected by early psychosis [27]. Despite an emerging evidence base for family interventions in early psychosis, and indeed a well-established evidence base for family interventions in chronic psychosis, the availability of these interventions in routine care is poor because of barriers at the level of services, clinician expertise, and the service user [71]. In addition, on entry into specialist first-episode services, carers often face an uncertain long-term prognosis for their relative and a process of recovery that extends well beyond the available 2-year period of care provided by state-supported specialist FEP care.

In this context of carer stress that may be prolonged, our objective was to exploit the convenience and accessibility afforded by the Internet to provide much needed support to family members affected by early psychosis. We had a specific focus on reducing carers’ stress by providing social support integrated with online psychoeducation and therapy. To the best of our knowledge, this is the first RCT designed to evaluate an online intervention for family members caring for a young person recovering from FEP.

Our control intervention includes the active specialised first-episode family care available to families during their relative’s episode of care within a specialist youth mental health service. The specific components include carefully targeted psychoeducation and support that are made available to all ‘first-episode families’ allocated to the treatment arm. For especially complex cases more intensive interventions are available, e.g. where there are severe behavioural disturbances [72]. Our control comparison condition is consistent with current treatment guidelines for early psychosis [72] and ensures the trial results will have the potential to inform whether the Altitudes intervention should be added to the provision of specialist care.

In addition to Altitudes being superior to STAU, our expectation is that we will find that Altitudes provides a cost-effective system of support beyond the short period of formal specialist care.

In addition to our novel online intervention, the current trial will provide highly novel data pertaining to the real-world experiences of carers. As recently argued by Lobban and Barrowclough, the currently available evidence for the interpersonal experiences of carers is derived from retrospective self-report and laboratory-based observations [73]. Measuring the everyday experiences of carers in (near) real time, as outlined in our procedure, will significantly enhance the understanding of carer and client outcomes and will drive the next generation of real-time interventions.

In conclusion, this is the first RCT to evaluate an online intervention for carers of young people recovering from FEP. It has the potential to provide support for a highly novel, accessible, and cost-effective intervention to reduce stress and improve wellbeing in carers who are providing support to young people at a critical phase in their recovery from psychosis.

### Trial status

As of September 2016, 75 participants have been randomised.

## Additional file

Additional file 1: The completed SPIRIT Checklist is available as an additional file. (DOC 122 kb)

## Abbreviations

CBT: Cognitive behaviour therapy
cRCT: Cluster randomised controlled trial
CRF: Case Report Form
DSM-IV-TR: *Diagnostic and Statistical Manual of Mental Disorders*, *fourth edition*, (*Text Revised*)
EPPIC: Early Psychosis Prevention and Intervention Centre
FEP: First-episode psychosis
HPA: Hypothalamic-pituitary-adrenal
MMRM: Mixed-model repeated measures
MOST: Moderated online social therapy
OYH: Orygen Youth Health
PSS: The Perceived Stress Scale
QALYs: Quality-adjusted life years
QoL: Quality of life
RA: Research assistant
RUQ: Resource Use Questionnaire
SEMA: Smartphone Ecological Momentary Assessment
STAU: Specialist treatment as usual
SWLS: Satisfaction With Life Scale
